# Sentinel lymph node identification in early stage ovarian cancer: is it still possible after prior tumor resection?

**DOI:** 10.1186/s13048-021-00887-w

**Published:** 2021-10-13

**Authors:** Pim Laven, Roy Kruitwagen, Petra Zusterzeel, Brigitte Slangen, Toon van Gorp, Jochem van der Pol, Sandrina Lambrechts

**Affiliations:** 1 Department of Obstetrics and Gynecology, Maastricht University Medical Center, Maastricht, The Netherlands; 2 GROW – School for Oncology and Developmental Biology, Maastricht University Medical Center, Maastricht, The Netherlands; 3 Department of Obstetrics and Gynecology, Radboud University Medical Centre, Nijmegen, The Netherlands; 4 Present address: Department of Obstetrics and Gynecology, Leuven University Medical Centre, Leuven, Belgium; 5 Department of Radiology Nuclear Medicine, Maastricht University Medical Center, Maastricht, The Netherlands

**Keywords:** Ovarian cancer, Sentinel lymph node

## Abstract

**Objective:**

Sentinel lymph node (SLN) detection in ovarian cancer is feasible when tracers are injected before the pathological ovary is resected. This study aims to investigate whether the SLN identification is also feasible in patients whose ovarian tumor has already been resected with injection of the tracer into the ovarian ligaments stumps, i.e. in the event that a frozen section confirms malignancy.

**Methods:**

Patients who underwent laparotomy with frozen section confirming an ovarian malignancy, and those who underwent a second staging laparotomy after prior resection of a malignant ovarian mass, were included. Blue dye and a radioactive isotope were injected in the stumps of the ligamentum ovarium proprium and the ligamentum infundibulo-pelvicum. After an interval of at least 15-min, the sentinel node(s) were identified using either the gamma-probe and / or blue dye.

**Results:**

A total of 11 patients were included in the study, the sentinel node (SLN) procedure was completed in all 11 patients. At least one SLN was identified in 3 patients, resulting in a rather low detection rate of 27,3%.

**Conclusion:**

In this study we showed that SLN procedure after (previous) resection of the tumor seems inferior to detect sentinel nodes when compared to injection of the tracer in the ovarian ligaments before tumor resection.

**Trial registration:**

NCT02540551

## Introduction

The International Federation of Gynecology and Obstetrics recommends surgical staging in patients with clinical early-stage epithelial ovarian cancer (EOC) including pelvic and para-aortic lymphadenectomy to detect lymph node metastases. There is 14% (range 6.1–29.6%) chance of finding lymph node metastases, implicating pathological advanced stage disease with an indication for adjuvant chemotherapy [1]. The more lymph nodes are removed, the higher the likelihood of detecting metastases [2]. Although complete pelvic and para-aortic lymphadenectomy has been shown to detect up to 250 lymph nodes, radical lymphadenectomy has been associated with serious potential morbidity [3–5] and there is therefore a great difference in the extent of lymph node dissection between different centers [6–8].

The concept of sentinel lymph node (SLN) surgery is widely used in different tumor types to assess whether the cancer has spread to the first lymph node in the lymph drainage pathway, called the SLN. The absence of cancer cells in the SLN is associated with a high probability that the cancer has not spread to other lymph nodes. The SLN technique has proven effective in the staging of various types of cancer, including breast cancer, malignant melanoma, and vulvar cancer [9–11]. In these tumor types the SLN technique has replaced the systematic performance of complete lymphadenectomy, reducing associated comorbidities such as lymphedema, and allowing to detect micro metastases after ultra-staging of the SLN.

Several studies have described the feasibility of the SLN technique in patients with early-stage EOC [12–24]. Most recent studies utilized a method in which a tracer is injected into the ovarian ligaments prior to tumor resection, with nearly all of these studies reporting very high detection rates (71–100%) of at least one SLN. If intraoperative pathologic examination of the frozen section confirms an ovarian malignancy, lymph node resection is performed. If the result of the frozen section shows a benign or borderline tumor, staging is not required and tracer injection prior to resection would have been unnecessary. Unfortunately, pre-operative techniques to predict ovarian malignancy based on tumor markers and ultrasound (risk of malignancy index (RMI), International Ovarian Tumor Analysis (IOTA) models) are not performant enough to avoid surgery of benign adnexal masses [25, 26], resulting in needless injection of (radioactive) tracer in case of benign or borderline pathology.

The main objective of this study was to investigate whether the SLN mapping technique is applicable to patients who have already undergone ovarian tumor resection. Using this technique, the tracer is injected into the stumps of the ovarian ligaments if either the result of a frozen section confirms malignancy, or definitive pathology confirms malignancy and a second surgical procedure is performed.

## Materials and methods

### Patients

Patients who underwent laparotomy with frozen section for a pelvic mass suspicious for malignancy and those who underwent a second staging laparotomy after prior resection of a malignant ovarian mass at Maastricht University Medical Centre or Radboud University Medical Centre Nijmegen were included in this study. All patients provided fully informed written consent prior to study enrolment, and the study protocol was approved by the local ethics committee (approval number: NL53246.068.15). Patients were excluded if they had undergone previous vascular surgery of the aorta, caval vein, and/or iliac vessels; if they had undergone previous lymphadenectomy or lymph node sampling in the iliac or para-aortal region; if they had a history of malignant lymphoma or malignant tumor in the abdominal cavity; if they had experienced a previous allergic reaction to blue dye or human albumin; or if they were pregnant or lactating.

### SLN procedure

**Fig. 1:**
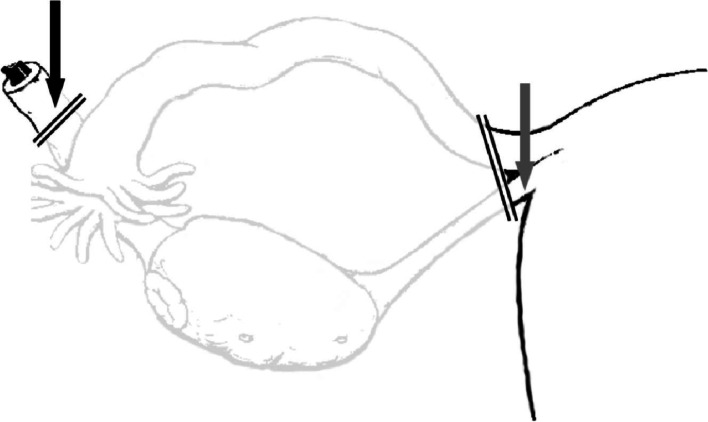
Location of injection of tracers. Tracers were injected on the ventral and dorsal sides of both ligament remains. Black arrow = remnant of infundibulo-pelvic ligament. Grey arrow = remnant of the ovarian ligament (proper ovarian ligament)

Patients with high suspicion of a malignant ovarian tumor received general anesthesia. After median laparotomy, the ovarian tumor was removed and sent to the pathologist for frozen section analysis. In case of a benign or borderline result, SLN detection was not attempted. If the frozen section showed malignancy, four aliquots, each containing 0.2 ml patent blue dye and 0.15 ml (20 MBq) of radioactive 99mTc-nanocolloid (Nanocoll®; GE Healthcare, Eindhoven, the Netherlands), were injected into the dorsal and ventral sides of the remains of the ligamentum ovarium proprium and the ligamentum infundibulo-pelvicum (lateral side) just below the peritoneum (Fig. 1). After an interval of at least a 15 min, the retroperitoneal space of the pelvic and para-aortic regions was opened, and the presence of SLN(s) was examined using the gamma-probe and/or visually (blue dye). The surgeon recorded the number and location of the resected SLNs. Twelve locations were assessed: the upper and lower para-aortic regions, the upper and lower inter-aortocaval regions, the upper and lower para caval regions, the right and left common iliac regions, the right and left external iliac regions, and the right and left obturator fossa regions. After removal of identified SLNs, the location was re-examined with the gamma probe for the presence of other lymph nodes containing more than 10% of the activity in the SLN. After this procedure with or without detection of SLNs, the complete standard staging procedure was performed, including a comprehensive random lymph node sampling of the bilateral pelvic and para aortal/caval region, hysterectomy, resection of the contralateral adnex and standard peritoneal biopsies.

The procedure performed in patients undergoing a second surgical staging procedure after resection of the ovarian malignancy was identical, starting with the injections of four aliquots of blue dye and radioactive tracer into the remnants of the ligaments.

### Histopathology

SLNs and non-SLNs were examined separately. Non-SLNs were cut into single sections and stained with hematoxylin and eosin (H&E), according to the standard protocol for lymph node examination. SLNs were cut into 2 mm sections for H&E staining. H&E-negative SLNs in the first section were further cut into 0.5 mm sections and examined for the presence of micro metastases (0.2 mm). At each step, the sections were immunohistochemically stained with antibody to cytokeratin MNF-116.

### Sample size calculation

In a previous study, the rate of detection of hotspot(s) following injection of radioactive tracers and a 15 min waiting period before resecting the adnexal mass was 100% [15]. Based on these results, a sample of 20 evaluable patients was considered large enough to determine whether identification of SLNs is feasible (detection rate of at least 50%) following a salpingo-oophorectomy and injection of tracers into the remnants of the ovarian ligaments. An interim analysis was planned after the evaluation of the first ten patients.

## Results

The interim analysis was performed after the inclusion of eleven patients (the last two patients were included within 1 week). Patient characteristics are shown in Table 1. In three patients only, at least one SLN was identified (27, 95%CI 10–54%). Although the CI reached just above 50%, this low percentage prompted us to terminate the study prematurely.

**Table 1:** Patient characteristics

Number of patients	11
Median age	57 (44–79)
Pre- or post menopause	
● Post	7 (63,6%)
● Pre	3 (27,3%)
● Unknown	1 (9,1%)
Surgery	
● Frozen section	8 (72,7%)
● 2nd surgery	3 (27,3%)
Frozen section malignant	8 (100%)
Final Pathology	11 (100%)
FIGO Stage	
● 1A	6 (54,6%)
● 1B	0
● 1C	4 (36,3%)
● 2A	0
● 2C	1 (9,1%)
Tumor type	
● *Clear cell*	5 (45,5%)
● *Serous*	1 (9,1%)
● *Mucinous*	2 (18,2%)
● *Endometroid*	3 (27,2%)

None of the patients experienced any allergic or adverse reactions. The SLN procedure was performed on the left side in six patients (54.4%), on the right side in four patients (36.4%), and on both sides in one patient (9.1%). In eight patients, the SLN procedure was performed during the primary surgical procedure after the frozen section showed malignancy. In the remaining three patients, the SLN technique was performed during a second surgical procedure between 5 and 8 weeks after primary surgery. In all three cases where the SLN was successfully detected, the SLN technique was performed during primary surgery.

### SLN detection and location after injection

In three out of eleven patients SLN’s were identified with the γ-probe 15 min after the injection of the radioactive tracer (Table 2). In two of these patients, one SLN each was identified on the ipsilateral side, the para-aortic, and the paracaval region. In the remaining patient, two SLNs were found, one in the inter-aorto-caval region and the other close to the common iliac artery on the contralateral side. No SLNs could be identified based on blue colorization. None of the SLNs were positive for metastasis. Furthermore, none of these three patients had lymph node metastases in the non-SLNs, which were resected to complete the staging procedure. Of the eight patients lacking SLN, one had a lymph node metastasis in a non-SLN.

**Table 2:** Sentinel nodes fou

Patient	Tumor side	Number of SLN	Histology during surgery	Location SLN	Histology after surgery	Metastases in the SLNs	Metastases in non-SLNs
1	Right	1	At least borderline	Paracaval low right	Mucinous	No	No
2	Left	1	Mixed	Para aortal low left	Clear cell	No	No
3	Left	2	Clear cell	Interaorta-caval, common iliac artery right	Clear cell	No	No

## Discussion

Many studies have evaluated the feasibility of detecting SLNs by injecting tracer(s) into the mesovarium and/or ovarian ligaments. Tracer injection prior to tumor resection and examination by frozen sections resulted in very high detection rates (87.5–100%) of at least one SLN [12–22]. The present study showed that injection of tracer into the remains of both ovarian ligaments after resection of the pathological adnex resulted in a low rate of SLN detection (3 out of 11 patients, 27%; 95%CI 10–53%). Too reach a detection rate of more than 50% as noted in the sample size calculation, we would have had to add at least 5 more patients with a positive node, which did not seem plausible in the current study design based on the results of the first 11 patients. For a detection rate comparable to the literature (90%) [12–22] we would even have had to include 69 more patients with a positive node. Because of these results, the study was terminated prematurely and we concluded that the identification of the sentinel node is not efficient after prior resection of the adnexal mass.

Although the study population in our study was small, the SLN detection rate was disappointingly low. To date, only three (two of the same research group) other studies assessed the detection of SLNs following the injection of tracer into the remnants of the ovarian ligaments after the adnexa were resected, either during the same or a subsequent surgical procedure [21, 25, 26].

In the study of Lago et al. the detection rate was 100% (10 out of 10 patients), being much higher than the 27% detection rate in the present study [21]. They injected the tracer deep into the parametrium rather than superficially under the peritoneum (shown on a video which was provided as an appendix), and also used indocyanine green as a tracer. The injection of the tracer deep into the parametrium resulted in a remarkably high number of cases with a *pelvic* (non-para aortal) sentinel node (88%), while the three SLNs detected in our study were all located at the para-aortal/para-caval level. The same research group later published a phase II clinical trial named the SENTOV study enrolling 20 patients where again the number of *pelvic* sentinel lymph nodes was high (93%) [24]. This may suggest that injection of the tracer after resection of the ovary deep into the parametrium, results in tracing uterine instead of ovarian pelvic sentinel lymph nodes. In this context, it should be noted that in a previous examaning ovarian lymphatic drainage we showed that the two major lymphatic drainage pathways of the ovaries invariably run via the suspensory ligament (infundibulopelvic ligament) and the proper ligament of the ovaries (ovarian ligament), the latter also being part of the uterine lymphatic drainage [27]. This would mean that with respect to the ovary, the presence of a pelvic sentinel node depends on whether the lymphatic flow direction in the ligament is from the ovary to the uterus or vice versa. Our lower para-aortal detection rate (27%) when compared to Lago et al. (70 and 100% respectively) may be the result of alteration of the lymphatic drainage after resection of the ovary, which makes detection of the actual para-aortal sentinel node after prior resection less reliable.

We do not think that the conflicting results can be explained by the use of different tracers. Indocyanine green is although its widespread use still not certified for its use as a tracer, and is particularly elegant for laparoscopy, which is in the setting of large adnexal masses warranting spill free resection not always possible. Furthermore, the advantage of ICG over radio-active technetium lies especially in the advantage of avoiding radio-activity, not in its detection performance since the effectiveness of technetium to detect the sentinel node is good, which was also the case in the SENTOV study (100% detection with 37 MBq radio-active technetium) and our previous research where good detection rates were reached with the same dose (20 MBq) radio-active technetium injected prior to resection of the adnex [15].

The third study examining the detection of SLNs following the injection of tracer into the remnants of the ovarian ligaments after the adnexa were resected, is a preliminary analysis of the first 31 patients enrolled in the SELLY trial, in 13 patients a secondary staging procedure was performed after an incidental diagnosis of ovarian cancer [23]. Using ICG as a SLN tracer they also reached a detection rate of 41,7% after resection of the adnexal mass, compared to 88,9% when immediate staging was performed. These results support our conclusion that the best results in identifying SLNs are obtained when the tracer is injected before the pathological ovary is resected.

The research group of Lago recently published a protocol for ultrastaging of the sentinel node, using section levels deepening every 200 μm – which is more detailed than the section levels every 500 μm used in the current study. The described technique allows an even more reliable detection of micro metastases [28].

The differences in injection tracers, doses and histopathological protocol used between the different studies urge the need for uniform standardization of the sentinel node technique in ovarian cancer, before it can successfully implemented in routine clinical practice.

## Conclusion

The SLN mapping technique has shown high detection rates of 87.5–100% when tracers are injected before resecting the adnexa [12–22]. This technique therefore seems feasible and may prevent lymphadenectomy. The present study showed that the rate of SLN detection after prior tumor resection was disappointingly low (27%). Injection of (radioactive) tracer is therefore not accurate to detect the sentinel node when after resection of the adnex, frozen sections show malignancy.

Based on these results, we support a standardized technique as proposed previously by Dell’Orto et al. [23], of one injection in both the suspensory and the infundibulopelvic ligament of the ovary in all patients with a high suspicion of an ovarian mass limited to the ovary. The use of indocyanine green as an alternative tracer for radio-active technetium seems promising but still needs to be examined on a larger scale especially in the case of laparotomy since its use for laparotomic staging of ovarian cancer has been reported in only a limited number of cases. Recognizing that a number of centers will not have near-infrared fluorescent technology, the use of radiocolloid and blue dye is an acceptable alternative. After a waiting time of 10–15 min after injection, the suspicious ovarian mass should be removed for frozen section analysis. If frozen section analysis confirms a malignancy, the staging procedure can start, including removal of the identified SLNs.

Using the proposed standardized technique, a protocol is designed to examine the accuracy and benefits of the SLN technique in an international multi-center collaborative study; based on the injection of tracers in the ovarian ligaments in patients with clinical early-stage ovarian cancer.

## Data Availability

The datasets used and/or analysed during the current study are available from the corresponding author on reasonable request.

## Abbreviations

SLN: Sentinel lymph node
EOC: Early stage ovarian cancer
